# Prospective multi‐institutional study of library‐based adaptive radiotherapy for cervical cancer: Evaluation of plan‐of‐the‐day selection and population analysis

**DOI:** 10.1002/acm2.70356

**Published:** 2025-11-18

**Authors:** Delphine Lebret, Caroline Lafond, Julie Leseur, Anaïs Barateau, Diane Chan Sock Line, Karine Peignaux, Nathalie Mesgouez‐Nebout, Magali Le Blanc‐Onfroy, Chantal Hanzen, Nedjla Allouache, Sophie Renard‐Oldrini, Florence Le Tinier, Renaud De Crevoisier, Antoine Simon

**Affiliations:** ^1^ University of Rennes, CLCC Eugène Marquis, INSERM, LTSI – UMR 1099 Rennes France; ^2^ Department of Radiation Oncology CLCC Eugène Marquis Rennes France; ^3^ Department of Radiation Oncology CLCC Georges‐François Leclerc Dijon France; ^4^ Institut de Cancérologie de l'Ouest Angers France; ^5^ Institut de Cancérologie de l'Ouest Saint‐Herblain France; ^6^ CLCC Henri Becquerel Rouen France; ^7^ CLCC François Baclesse Caen France; ^8^ Centre Alexis Vautrin Nancy France; ^9^ CLCC Oscar Lambret Lille France

**Keywords:** adaptative radiotherapy, CBCT segmentation, cervical cancer, plan‐of‐the‐day, treatment plan library

## Abstract

**Purpose:**

Plan‐of‐the‐day (PoD) adaptive radiation therapy (ART) is based on a library of treatment plans, with 3D daily imaging guiding the plan selection. In a phase II multi‐institutional trial of cone‐beam CT (CBCT)‐guided PoD‐ART for locally advanced cervical carcinoma (LACC), this study aimed at evaluating the PoD selection, its geometric and dosimetric impact and characterizing a sub‐population of patients associated with dosimetric improvement from ART.

**Material and methods:**

For 49 cervical cancer patients, three planning CT scans [empty bladder (EB), intermediate bladder (IB) and full bladder (FB)] were acquired to generate a treatment plan library. A dose of 45 Gy was prescribed to the planning target volume in 25 fractions. Daily CBCT were acquired to visually select the best plan in the library (Manual‐ART strategy). A deep learning model was used to segment daily clinical target volume (CTVt) and organs‐at‐risk (OAR). Manual‐ART was compared to two strategies: (i) “Non‐ART” strategy (IB‐CT treatment plan only); (ii) PoD‐ART strategy selecting the PoD maximizing CTVt coverage (“Cov‐ART”). Geometrical and dosimetric coverages of daily CTVt and OAR were assessed. Decision trees were developed to predict the subpopulation of patients associated with dosimetric benefit from PoD‐ART.

**Results:**

The agreement in PoD selection between Manual‐ART and Cov‐ART was 63.5%. Compared to the Non‐ART strategy (D95%‐CTV: 43.6 ± 4.1 Gy), PoD‐ART significantly increased the dose to the target, with Manual‐ART achieving 44.0 ± 3.0 Gy and Cov‐ART with 44.1 ± 2.0 Gy. Decision trees using IB‐CT plan and first two treatment fractions correctly classified 85.4% and 93.8% of patients as benefiting or not from PoD‐ART.

**Conclusions:**

In PoD‐ART for LACC, selected treatment plans by the radiation oncologist had 63.5% concordance with treatment plans maximizing target coverage. PoD‐ART increased dose to target, without compromising dose to OARs, with the largest benefit observed in a sub‐population identifiable after two treatment fractions.

## 1 INTRODUCTION

Locally advanced cervical cancer (LACC) is commonly treated with external beam radiation therapy (EBRT) and chemotherapy, followed by brachytherapy. While intensity‐modulated radiation therapy (IMRT) can reduce normal tissue toxicity,^1^, ^2^ it faces challenges from significant and large anatomical variations within the pelvic area.^3^, ^4^ Indeed the position and shape of the clinical target volume (CTVt) depend on bladder and rectal fillings. Average uterine displacements of 1–16 mm in the antero‐posterior and 15–24 mm in the supero‐inferior directions between fractions have been reported.^5^, ^6^, ^7^ In addition, tumor shrinkage during treatment was observed with an average reduced volume by 46% of the primary gross tumor volume (GTV) after three treatment weeks.^8^ These anatomical variations can lead to a decrease in the dose received by the CTVt and increase the dose received by organs at risk (OARs).^3^, ^9^

To compensate for these anatomical variations, Plan‐of‐the‐Day adaptive radiation therapy (PoD‐ART) strategies have been proposed.^10^, ^11^, ^12^, ^13^ These strategies consist in building a library including several treatment plans based on several planning computed tomography (CT) scans, acquired with different bladder fillings. At each treatment fraction, based on daily imaging (typically kilovoltage cone‐beam CT [CBCT]), the treatment plan is then selected among those available in the library (PoD). This approach allows for the customization of treatment plans based on patient‐specific anatomical changes. Among the existing studies of PoD‐ART strategies based on different bladder fillings,^10^, ^12^, ^13^, ^14^, ^15^, ^16^, ^17^, ^18^ only two evaluated the clinical implementation in terms of both feasibility and dosimetric evaluation.^11^, ^16^ In clinical practice, CBCT images are not routinely delineated due to challenges such as presence of gas, limited contrast, large deformations, which limits quantitative evaluation. Furthermore, most prior studies involve small number of patients (< 30).

To our knowledge, no previous multi‐institutional clinical study has combined geometric and dosimetric evaluation of PoD‐ART over the entire treatment course in a multi‐center setting. Additionally, the ability to predict an individual patient's benefit from adaptation remains unexplored, despite its potential to optimize treatment planning and resource allocations. The aim of this study was first to evaluate the benefit of PoD‐ART for LACC in a prospective multi‐institutional clinical trial framework. The evaluation was performed with geometric and dosimetric endpoints, as well as an assessment of the radiation oncologist's treatment plan selection.

The selection of the plan of the day by the radiation oncologist was firstly evaluated by comparing the manually selected PoD (Manual‐ART) with the plan maximizing geometric coverage (Cov‐ART). PoD‐ART was also compared to: (i) standard treatment (one plan based on CT with intermediate bladder volume); (ii) treatment plan maximizing the geometric coverage of the daily CTVt by an automatic selection (Cov‐ART). An automatic segmentation method based on a deep‐learning network was used to segment CBCT images, and then the geometric and dosimetric impacts of these strategies were evaluated. Moreover, the prediction of the individual benefit of PoD‐ART was assessed with a decision tree using patient data and images acquired at planning and during the first week of treatment.

## 2 MATERIALS AND METHODS

### 2.1 Data acquisition and experimental settings

This study was based on data from 49 locally advanced cervical cancer patients that have been included in a phase II PoD‐ART multi‐center clinical trial (ARCOL) conducted across seven French clinical centers between April 2017 and April 2022. According to the classification of the International Federation of Gynecology and Obstetrics (FIGO),^19^ the stage of the patients was as follows: IB1, *n* = 3; IB2, *n* = 20; IIA, *n* = 2; IIB, *n* = 23; III, *n* = 1. Additionally, histological analysis revealed squamous cell carcinoma for 38 patients, adenocarcinoma for 10 patients, and other histological type for one patient. All patients provided signed informed consent.

Protocol deviations were systematically classified as critical, major or minor based on their impact on patient safety and data integrity. The analysis excluded those with major deviations (e.g., inadequate FIGO stage, missing CT‐scans) or incomplete treatment. These deviations were regularly reviewed by a committee (composed of physicians and methodologists), which also determined if data exclusion and corrective actions were necessary.

A total of 158 CT scans and 1217 daily CBCT scans were collected. CBCTs came from seven centers: one center (Center 1, 18 patients) used XVI Elekta System and centers 2 through 7, comprising 8, 3, 3, 6, 10 and 1 patients, respectively, used OBI Varian systems.

All patients were treated with a combination of external beam radiation therapy (EBRT) and pulse‐dose‐rate brachytherapy (PDR‐BT). EBRT delivered a total dose of 45 Gy to the pelvic region, administrated at 1.8 Gy per fraction using IMRT or VMAT, along with concomitant weekly cisplatin (40 mg m^−2^). PDR‐BT was delivered according to the GEC‐ESTRO guidelines.^20^

**FIGURE 1:**
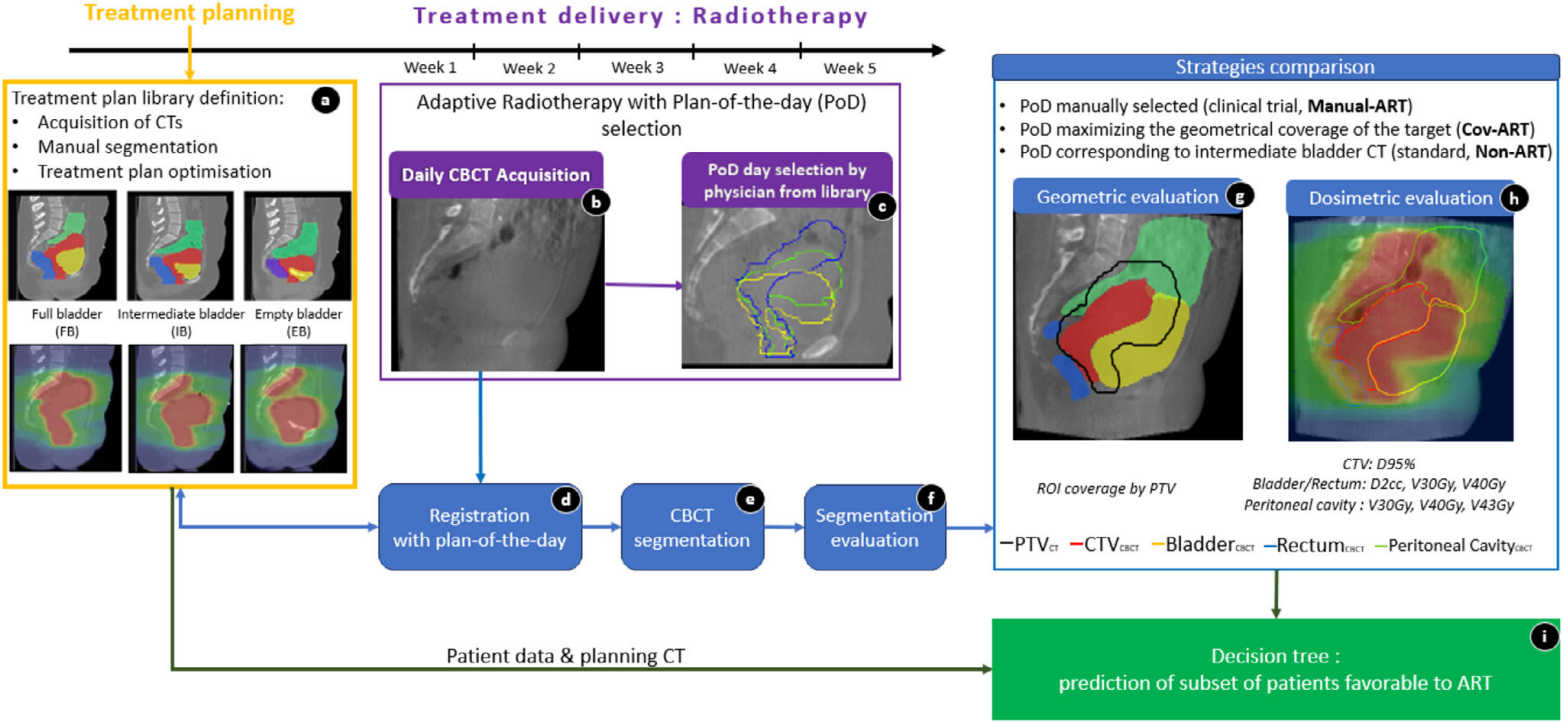
Workflow of the study with nine main steps: (a) Planning: acquisition of three planning CT scans with variable bladder volumes. (b) Acquisition of the daily CBCT image. (c) Selection of the Plan‐of‐the‐Day (PoD). (d) Bones registration between CBCT and PoD. (e) Automatic segmentation of CTVt, Bladder, Rectum and Peritoneal cavity. (f) Segmentation evaluation by an expert radiation oncologist. (g) Geometric evaluation and comparison of the three strategies. (h) Dosimetric evaluation and comparison of the three strategies. (i) Prediction of subset of patients favorable to ART using decision tree.

For each patient, three planning CT (pCT) scans were acquired with different bladder fillings: empty bladder (EB), intermediate bladder (IB), and full bladder (FB) resulting in a library of three treatment plans as shown in Figure 1a. For this purpose, patients drank 250 mL of water 1 h before the first CT scan (IB‐CT). For the second CT scan (FB‐CT), the patient drank 500 mL of water, followed by a 20 min waiting period. For the third scan (EB‐CT), they emptied their bladder beforehand.

To generate the patient‐specific treatment plan library, the clinical target volume including the primary tumor site (CTVt)—comprising the cervical tumor and uterus—and the regional lymphatics nodes (CTVn), as well as the organs‐at‐risk (bladder, rectum, and peritoneal cavity), were manually contoured on all three CT scans (EB‐CT, IB‐CT, FB‐CT) by expert radiation oncologists. The planning target volume (PTV) of each plan was defined by the primary tumor site with a 10 mm isotropic margins, and by the regional lymphatics nodes with 5 mm isotropic margins.

At each treatment fraction, a CBCT scan was firstly acquired. Based on this daily CBCT scan, the radiation oncologist selected the plan of the day (PoD), by visualizing the projection of the three planning CTVs on the daily CBCT (Figure 1c). The manual plan selection was performed by 16 radiation oncologists across the seven clinical centers. Radiation oncologists chose the most appropriate plan by visually assessing which PTV among the three treatment plans best encompassed the uterus while minimizing irradiation to OAR. If the uncertainty was considered too large (e.g. in the case of poor image quality), the intermediate bladder plan was selected per default as defined in the clinical trial. The perceived difficulty of the daily plan selection was also rated as easy, difficult, very difficult or other by the radiation oncologist. To harmonize plan selection across centers, a dummy run was performed: 16 oncologists reviewed 24 standardized CBCTs and selected the PoD as they would do in clinical practice.^21^

The workflow of this study is described in Figure 1. To simulate patient repositioning, each daily CBCT scan was rigidly registered with the CT corresponding to the plan of the day (Figure 1d). This registration was performed using Elastix software^22^ with a normalized correlation coefficient. The resulting rigid transformation was visually validated by assessing the alignment of bony structures between the registered images.

### 2.2 CBCT Segmentation using deep‐learning

As described in a previous study,^23^ a separate database consisting of 273 CBCT scans (XVI, Elekta) from 23 patients was used to train a deep learning model for the automatic segmentation of the main structures in CBCT scans: CTVt, bladder, rectum, and peritoneal cavity. To overcome the limited availability of contoured CBCT scans, the training set was augmented by incorporating two CT scans from the treatment plan library of each patient. The segmentation model was based on the deep learning method nnU‐Net^23^, ^24^ which has been demonstrated to be efficient for segmenting regions of interest (CTVt and OARs) in the pelvic region on CBCT scans.^23^ The segmentation model was trained using a 3D full‐resolution U‐Net architecture using PyTorch with parameters described in Zhang et al.^23^ The comprehensive evaluation of the model produced the following median Dice coefficients across the 272 images: 0.79 for CTVt, 0.84 for Bladder, 0.75 for Rectum and 0.81 for the peritoneal cavity. After training, the model was applied to the 1217 CBCT images of the 49 patients included in this study. Resulting segmentations were reviewed and assessed by an expert radiation oncologist, who categorized them into three quality levels: good segmentation, intermediate (minor errors, clinically acceptable for PoD selection) and poor (clinically unacceptable). CBCT scans associated with poor segmentations were excluded from the following analysis.

### 2.3 Geometric evaluation of the PoD selection

To evaluate the selection of PoD by radiation oncologist (“Manual‐ART” strategy), a geometric evaluation was firstly performed, as presented in Figure 1g. The endpoint was the relative coverage of the daily CTVt (CTV_CBCT_) by the CTVt of the selected plan (CTV_PoD_). This coverage was computed as follows (Equation 1):

(1) $$\mathrm{Co}v_{\mathrm{CTV}}=\frac{\left|\mathrm{CT}V_{\mathrm{CBCT}}\cap \mathrm{CT}V_{\mathrm{PoD}}\right|}{\left|\mathrm{CT}V_{\mathrm{CBCT}}\right|}$$

The PoD corresponding to the best geometrical coverage (highest value) of the daily CTVt was then identified. In the following, this approach is named “Cov‐ART”.

The manually selected PoDs were compared with the PoDs according to the Cov‐ART approach. Furthermore, the influence of the difficulty in manually selecting the PoD was investigated to determine whether it could explain the potential discrepancies between these strategies.

Moreover, to assess the ability of the selected treatment plans to spare the OARs, the coverage (as a percentage of volume) of each OAR (${\mathrm{ROI}}_{\mathrm{CBCT}}\in \left\{{\mathrm{Bladder}}_{\mathrm{CBCT}},{\mathrm{Rectum}}_{\mathrm{CBCT}},\mathrm{Peritoneal}\;{\mathrm{Cavity}}_{\mathrm{CBCT}}\right\}$) by the PTV of the selected PoD was computed. The lower were these values, the better was the PoD in terms of OAR sparing. This calculation was performed on all daily CBCT scans and defined as the normalized intersection between the selected PTV (${\mathrm{PTV}}_{\mathrm{CT}}\in \left\{{\mathrm{PTV}}_{\mathrm{EB}},{\mathrm{PTV}}_{\mathrm{IB}},{\mathrm{PTV}}_{\mathrm{FB}}\right\}$) and the corresponding organ of interest (Equation 2):

(2) $$\mathrm{Co}v_{\mathrm{ROI}}=\frac{\left|\mathrm{RO}I_{\mathrm{CBCT}}\cap \mathrm{PT}V_{\mathrm{CT}}\right|}{\left|\mathrm{RO}I_{\mathrm{CBCT}}\right|}$$

To evaluate the performance of the Manual‐ART strategy, it was compared to two other strategies that were simulated for each patient: (i) the “Non‐ART” strategy, where the PoD was systematically corresponding to intermediate bladder; (ii) the Cov‐ART previously defined. Coverages values from these strategies were compared using a nonparametric paired test (Wilcoxon signed‐rank test) with Bonferroni correction.

### 2.4 Dosimetric evaluation of the strategies

To enable daily dose evaluation, the dose matrix corresponding to the PoD of each strategy was applied to the CBCT, using the result of rigid registration, assuming invariance of the dose matrix.^25^ Dosimetric analysis comparing daily dosimetric indices was performed between the three strategies. The following indices were averaged across all treatment fractions: dose received by 95% of the CTVt volume (D95%‐CTV), maximal dose (D_2cc_) and volume receiving 30 and 40 Gy (V_30Gy_ and V_40Gy_) for bladder and rectum, as well as the volume of the peritoneal cavity receiving 30, 40 and 43 Gy (V_30Gy_, V_40Gy_ and V_43Gy_).

To assess the compliance to the prescribed dose to the CTVt, the number of treatment fractions for which the D95%‐CTV complied with the minimum threshold of 42.75 Gy (95% of the prescribed dose) was determined for each strategy.

### 2.5 Prediction of the subset of patients favorable to PoD‐ART

The dosimetric benefit of the Manual‐ART strategy over the Non‐ART strategy was assessed by computing the difference of D95%‐CTV (Δ_D95% _= D95%‐CTV_Manual‐ART_—D95%‐CTV_Non‐ART_) and of V_40Gy_ to the OARs represented by bladder, rectum and peritoneal cavity (Δ_V40Gy _ = $\frac{1}{3}\sum_{o\in \mathrm{Bladder},\mathrm{Rectum},\mathrm{Peritoneal}\;\mathrm{Cavity}}(V40Gy_{o,\mathrm{Manual}-\mathrm{ART}}-V40Gy_{o,\mathrm{Non}-\mathrm{ART}}$).

Based on the difference in Δ_D95%_ to CTVt and Δ_V40Gy_ to OARs, patients were subsequently grouped according to whether they benefited from Manual‐ART. To provide flexibility for clinical implementation across institutions with varying adaptive capabilities, thresholds were chosen to reflect both light and major dosimetric gains. A patient was considered as benefiting from Manual‐ART if any of the following criteria was met:

1. Target coverage: ART Improvement of D95% to the CTV: +1 Gy for the light dosimetric gain and +3 Gy for major dosimetric gain
2. OARs constraints: ART improvement of the mean V40Gy to bladder, rectum and bowel cavity: 5% for the light dosimetric gain favorable to ART and 20% for major dosimetric gain favorable to ART

A line was then considered between the two points corresponding to these criteria, resulting in the equation y = ‐5 + 5*Δ_D95%_ for the light dosimetric gain, and *y* = ‐20 + $\frac{20}{3}$ *Δ_D95%_ for major dosimetric gain. These lines, as shown in Figure 4, resulted in the separation of the three population groups. Patients below the red curve showed dosimetric gains to the target and/or OAR sparing with manual PoD selection, making them favorable for Manual‐ART (fav‐ART). The goal was to consider a scenario in which a standard treatment is firstly considered for all patients, and only patients favorable to ART are actually switched to ART. Statistics were thus computed to describe each subgroup using patient data (e.g., age, body mass index, FIGO stage, and fertility status) and imaging acquired at planning (the Sorensen‐Dice similarity coefficient^26^ between EB‐CT and FB‐CT) and CBCT images during the first week of treatment (Dice score between CTVt in IB‐CT and CTVt of the day, D95%‐CTV, V40Gy to bladder, rectum and peritoneal cavity). Significant intergroup differences were identified using the non‐parametric Wilcoxon rank‐sum test with Bonferroni correction.

Decision trees were modeled to classify patients into groups, by incorporating different numbers of treatment fractions in the model (from 0 to 5). The classification thresholds were automatically determined using the rpart package in R,^27^ based on the Gini index as the splitting criterion.^28^ This approach aims to maximize class separation by selecting the most discriminative thresholds from training data with ROC curve optimization. To ensure robustness and avoid overfitting in our dataset, we applied leave‐one‐out cross validation, where each case is iteratively held out for testing while the rest are used for model training. The final decision tree was selected based on its predictive performance and its use of the minimal number of treatment fractions.

## 3 RESULTS

### 3.1 Performance of segmentation by deep‐learning

A total of 1217 CBCT images were automatically segmented using the nnU‐Net method. Among the delineated contours, 21% were classified as good, 55.4% as intermediate, and 23.6% as poor. The latter were discarded from the following analyses, which were thus based on 930 treatment fractions.

Moreover, quality assurance analysis demonstrated no statistically significant difference in segmentation accuracy between Varian OBI and Elekta XVI systems (*ρ* > 0.05)

### 3.2 Geometric evaluation of the three strategies

#### 3.2.1 Selection of the PoD

**FIGURE 2:**
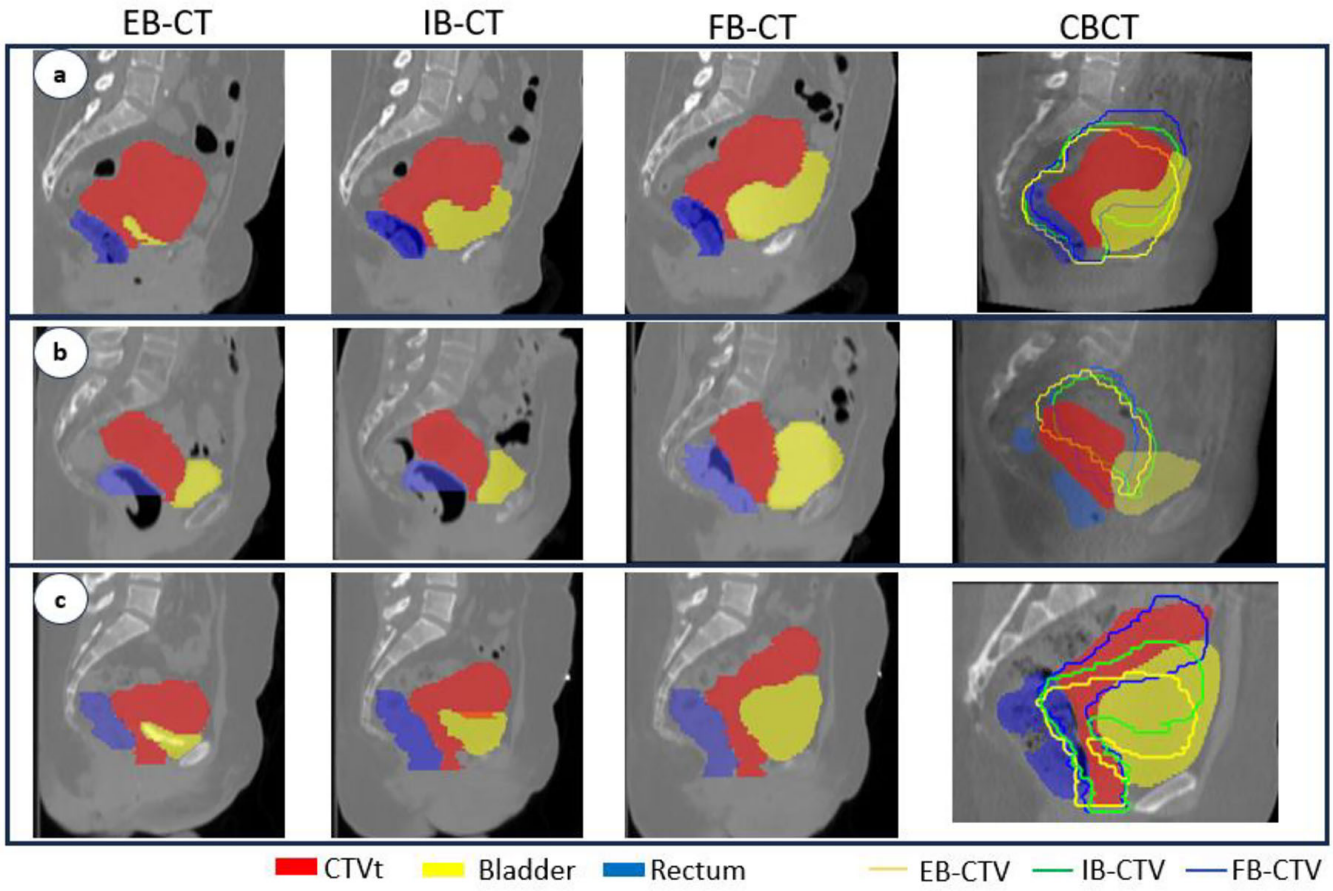
For each case: the three planning CTs (Empty bladder EB, Intermediate bladder IB and Full bladder FB) followed by the CBCT of the day with the daily CTVt (red) and CTVts from planning CT overlaid. Examples of cases: (a) Concordant PoD choice (FB plan); (b) Major discordant choice: CTVt located outside all three planning CTs (FB instead of EB); (c) suboptimal manual choices without justification (EB instead of FB).

Table 1 illustrates agreements between the Manual‐ART and the Cov‐ART strategies which represents the best PoD in terms of geometric coverage of the target. 63.5% (773 fractions) agreement was found between the Manual‐ART and the Cov‐ART strategies (Figure 2a). In the remaining 36.5% (444 fractions) of cases with discrepancies, 32.7% were moderate discrepancies, such as selecting the empty bladder plan instead of the intermediate bladder plan, or inversely. The remaining 3.8% (46 fractions) of discrepancies were important ones, involving full and empty bladder plans.

**TABLE 1:** Table of agreement between visually selected PoD (Manual‐ART) and optimal PoD maximizing geometric coverage of the target (Cov‐ART).

Agreement between the two strategies of plan selection					
	Cov‐ART (% of fractions)				
	Treatment plan based on CT with	Empty bladder	Intermediate bladder	Full bladder	Total
Manual‐ART (% of fractions)	Empty bladder	20.2	3.4	1.7	25.3
	Intermediate bladder	12.7	22.4	11.5	46.6
	Full bladder	2.1	5.1	20.9	28.1
	Total	35.0	30.9	34.1	100

*Note*: Concordance in green, minor discordance in yellow, major discordance in red.

In discordant cases between manual‐ART and Cov‐ART, three main situations were identified: (i) plans were nearly identical, making the choice subjective (Figure 2b); (ii) the daily CTVt was intermediate between two plans, leading to differing trade‐offs; (iii) the rationale for the selected plan could not be clearly determined (Figure 2c).

The reported difficulty of treatment plan selection by radiation oncologists was distributed as follows: 62.3% of the fractions were classified as easy, 24.9% as difficult, 10.8% as very difficult, and 2% were reported as unknown.

A positive association was obtained between the difficulty of the plan selection by the radiation oncologist and the level of agreement. Manual choices rated as easy corresponded to higher concordance rates between the radiation oncologist and the automated plan selection (69.6% of concordance, 2.4% of major discordances). In contrast, choices rated as difficult were associated with lower concordance rates (57.9% of concordance, 5.1% of major discordances).

#### 3.2.2 Geometric evaluation of the PoD of the three strategies

**FIGURE 3:**
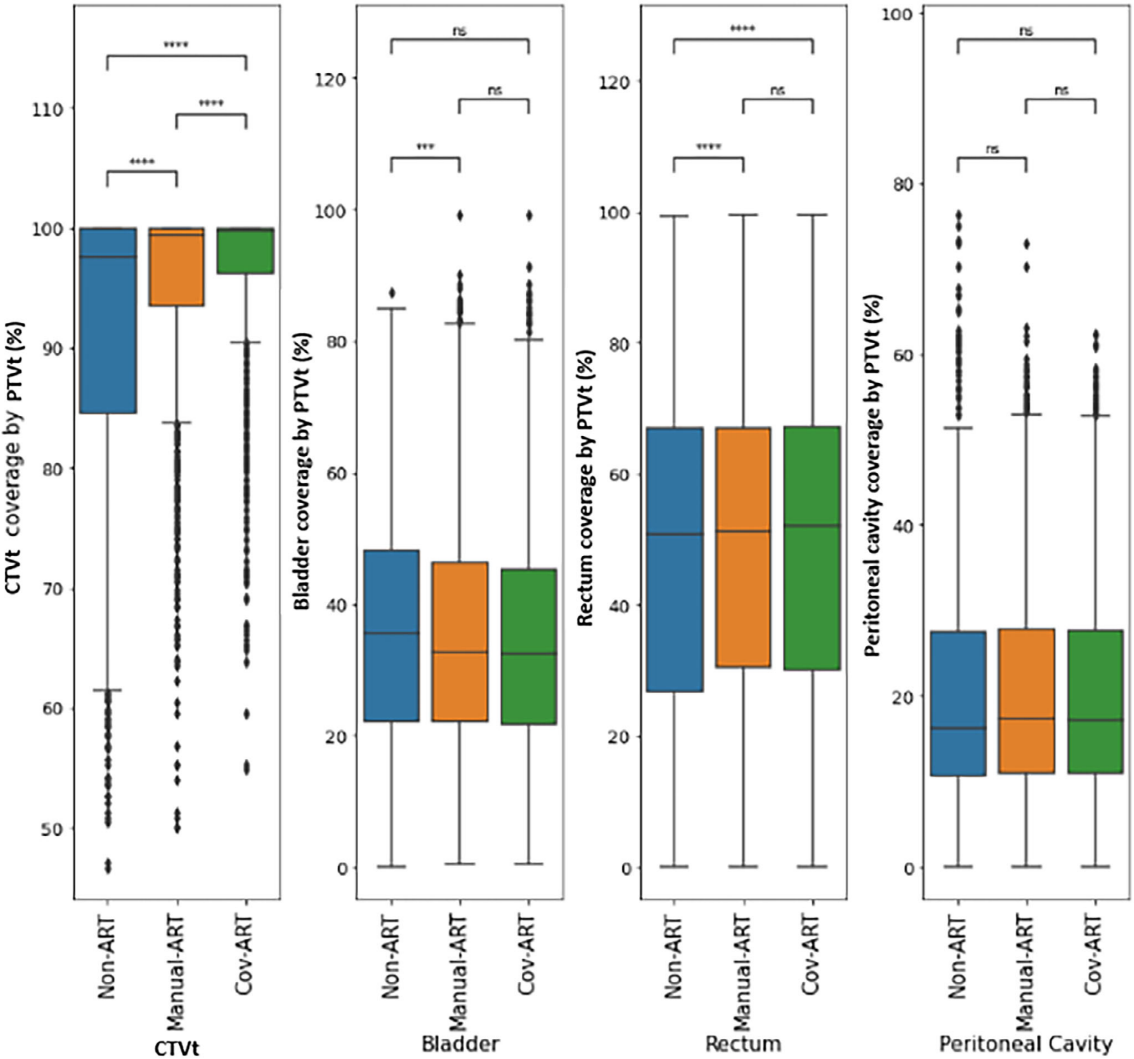
CTVt and OAR geometric coverage by the PTV for all treatment fractions, and for the three strategies (ns: *p* > 0.05, *p < 0.05, **p < 0.01, ***p < 0.001, ****p < 0.0001).

Figure 3 illustrates the geometric coverage of the CTVt and OARs by the PTV for each strategy over the entire treatment. Both Manual‐ART and Cov‐ART strategies led to a significant increase in geometric coverage (*p* < 0.001) of the daily CTVt compared to the Non‐ART strategy. The median geometric coverage of the target was significantly higher with adaptive strategies, reaching 99.4% for manual plans and 99.8% for Cov‐ART plans, compared to 97.6% for Non‐ART plans. The minimum value of CTVt coverage improved from 46.7% with Non‐ART to more than 50.0% with adaptive strategies (50.0% for Manual‐ART and 54.9% for Cov‐ART, Figure 3).

A slight significant decrease in bladder coverage was observed with Manual‐ART compared with Non‐ART and Cov‐ART (*p* < 0.001). However, Cov‐ART and Manual‐ART strategies led to significant increases in rectum coverage (*p* < 0.001) when compared to the Non‐ART strategy.

### 3.3 Dosimetric evaluation of the three strategies

Table 2 presents the dosimetric values of each strategy for CTVt and OARs. Both adaptive strategies (Manual‐ART and Cov‐ART) showed a significant dosimetric increase to the CTVt in terms of D95%‐CTV (*p* < 0.001) compared to the non‐adaptive strategy (44.0 and 44.1 vs. 43.6 Gy). Specifically, the minimum D95%‐CTV increased from 17.7 Gy with Non‐ART strategies to more than 20 Gy with adaptive strategies (20.5 Gy with Manual‐ART and 20.9 Gy with Cov‐ART).

**TABLE 2:** CTVt and OAR dosimetric values for all strategies.

Dosimetric indices to CTVt and OARs following strategies: median (min‐max)										
	CTVt	Bladder			Rectum			Peritoneal Cavity		
	D_95%_ (Gy)	D_2cc_ (Gy)	V_30Gy_ (%)	V_40Gy_ (%)	D_2cc_ (Gy)	V_30Gy_ (%)	V_40Gy_ (%)	V_30Gy_ (%)	V_40Gy_ (%)	V_43Gy_ (%)
**Non‐ART**	43.6 (17.7‐44.8)	45.7 (37.6–55.1)	74.0 (6.6–100.0)	45.4 (1.2–90.7)	44.9 (36.7–47.2)	92.3 (17.4–100.0)	59.1 (1.3–99.6)	49.9 (4.3–96.8)	30.4 (0.1–85.6)	23.0 (0.0–79.1)
**Manual‐ART**	44.0^a^ (20.5–45.1)	45.7^c^ (35.9–55.1)	73.3^c^ (16.2–100.0)	44.1^c^ (0.5–100.0)	44.9^c^ (36.7–49.8)	94.3^c^ (17.4–100)	60.4^c^ (1.3–100.0)	50.3^c^ (5.7–94.8)	30.9^c^ (0.5–84.2)	23.5^c^ (0.1–77.4)
**Cov‐ART**	44.1^a,b^ (20.9–45.1)	45.8^c^ (33.9–55.1)	73.7^c^ (23.7–100.0)	44.5^c^ (0.1–100.0)	45.0^c^ (30.9–50.7)	94.9^c^ (8.6–100)	62.1^c^ (0.1–100.0)	51.1^c^ (5.7–100.0)	30.9^c^ (0.5–83.7)	23.7^c^ (0.1–76.3)

^a^ means *p* < 0.001 between Non‐ART versus ART strategy.

^b^ means *p* < 0.001 between the both ART strategies.

^c^ means *p* > 0.05 between Non‐ART versus ART strategy and between the both ART strategies.

The D95% to the CTVt increased from the Manual‐ART and Cov‐ART strategies, with increases of 0.9% and 1.1%, respectively. The dose delivered by the adaptive strategies ensured compliance with the minimum D95%‐CTV required by the dose constraints (42.8 Gy), from 25% with Non‐ART to 14% with Manual‐ART and 10% with Cov‐ART.

For OARs, no significant differences were observed among the three strategies for maximal doses (V_40Gy_ and V_30Gy_). Similarly, no significant differences were found for all dosimetric indices in the peritoneal cavity.

### 3.4 Predicting the subset of patients favorable to ART

**FIGURE 4:**
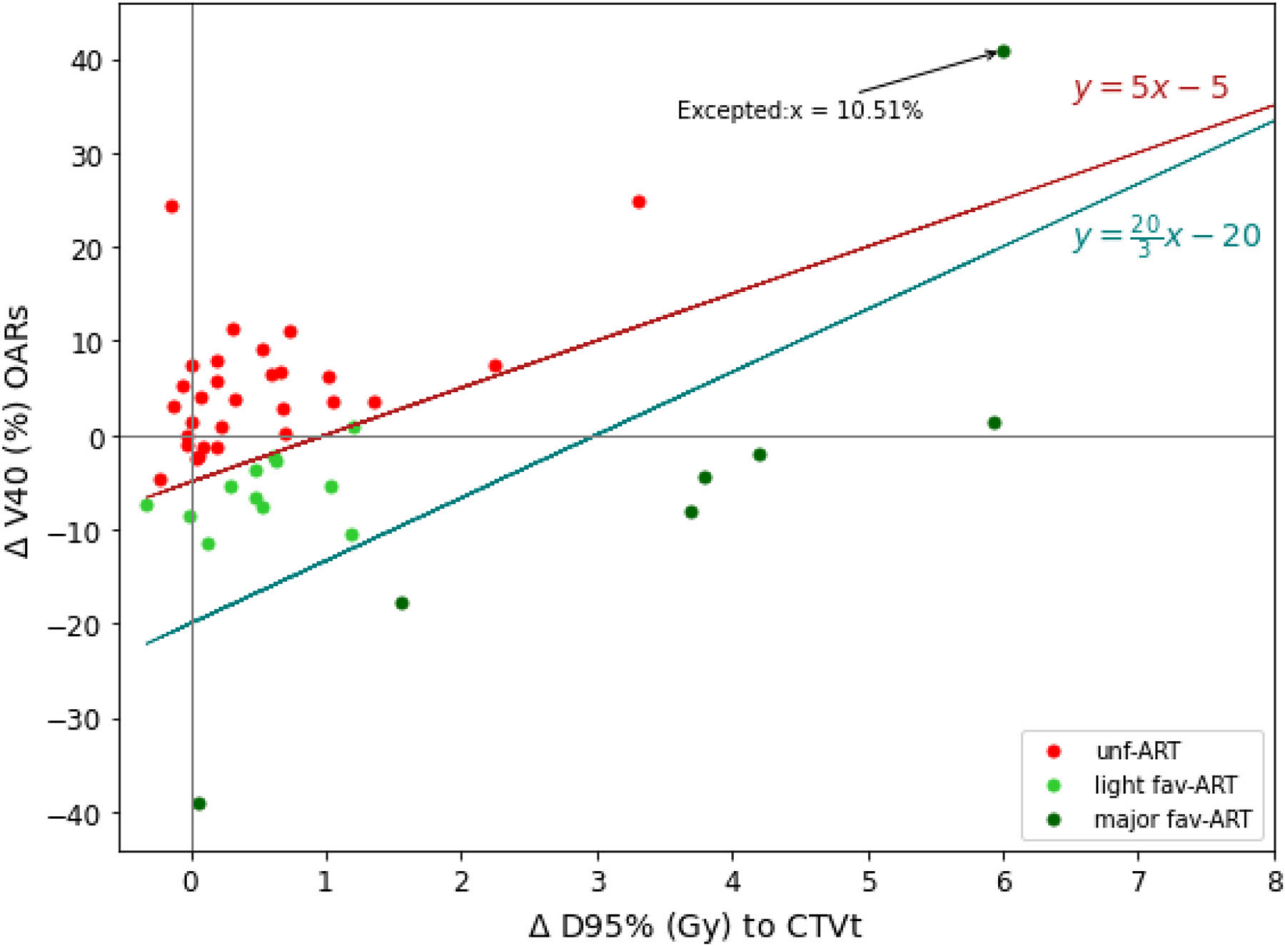
Representation of groups unf‐ART (red), light fav‐ART (light green) and major fav‐ART (dark green) separated by respectively red and blue lines, according to their dosimetric benefit at the CTVt (Δ_D95%_), and difference of volume receiving 40 Gy to OARs (Δ_V40Gy_), with Manual‐ART compared to Non‐ART.

Patients above the red curve represented those with target degradation and/or increased OAR dose when using Manual‐ART, making them unfavorable for the Manual‐ART strategy (unf‐ART). The prediction of the corresponding group (fav‐ART or unf‐ART) for each patient was studied by considering descriptors computed at planning and during the first week of treatment, using the three planning CT‐scans, but only the treatment plan corresponding to the standard treatment (IB). Figure 4 shows that 37% of patients had both V40Gy sparing of OARs (Δ_V40Gy_ < 0%) and increase in CTVt dose with Manual‐ART (Δ_D95%_ > 0 Gy). Each point represents a patient with the gain in D95% to the CTVt and the difference in V40Gy to the OARs between Manual‐ART and Non‐ART. Moreover, 13% of patients had a significant dose increase at the CTVt with a small dose increase on OARs. Therefore, the red straight line (*y* = ‐5 +5*Δ_D95%_) separates the two groups into unf‐ART and fav‐ART: 38% of patients were in the fav‐ART group (green) and 62% in the unf‐ART group (red). The blue line (*y* = ‐20 + $\frac{20}{3}$ *Δ_D95%_) further separates patients with greater dosimetric benefit, distinguishing the light fav‐ART group (light green) from the major fav‐ART group (14% of the patients, dark green).

Statistical analysis of the two‐group comparison (unf‐ART and fav‐ART) identified three significant variables: the D95%‐CTV to the target, the V40 Gy to the rectum on the IB‐CT planning and the mean dose of D95%‐CTV received during the first and second fractions treatment based on the intermediate bladder plan (IB‐CT). For the decision tree with major dosimetric gain, the significant variables were also the D95%‐CTV to the target during the first and second treatment fractions, as well as the D95%‐CTV to the target and the V40 Gy to the bladder on IB‐CT planning.

**FIGURE 5:**
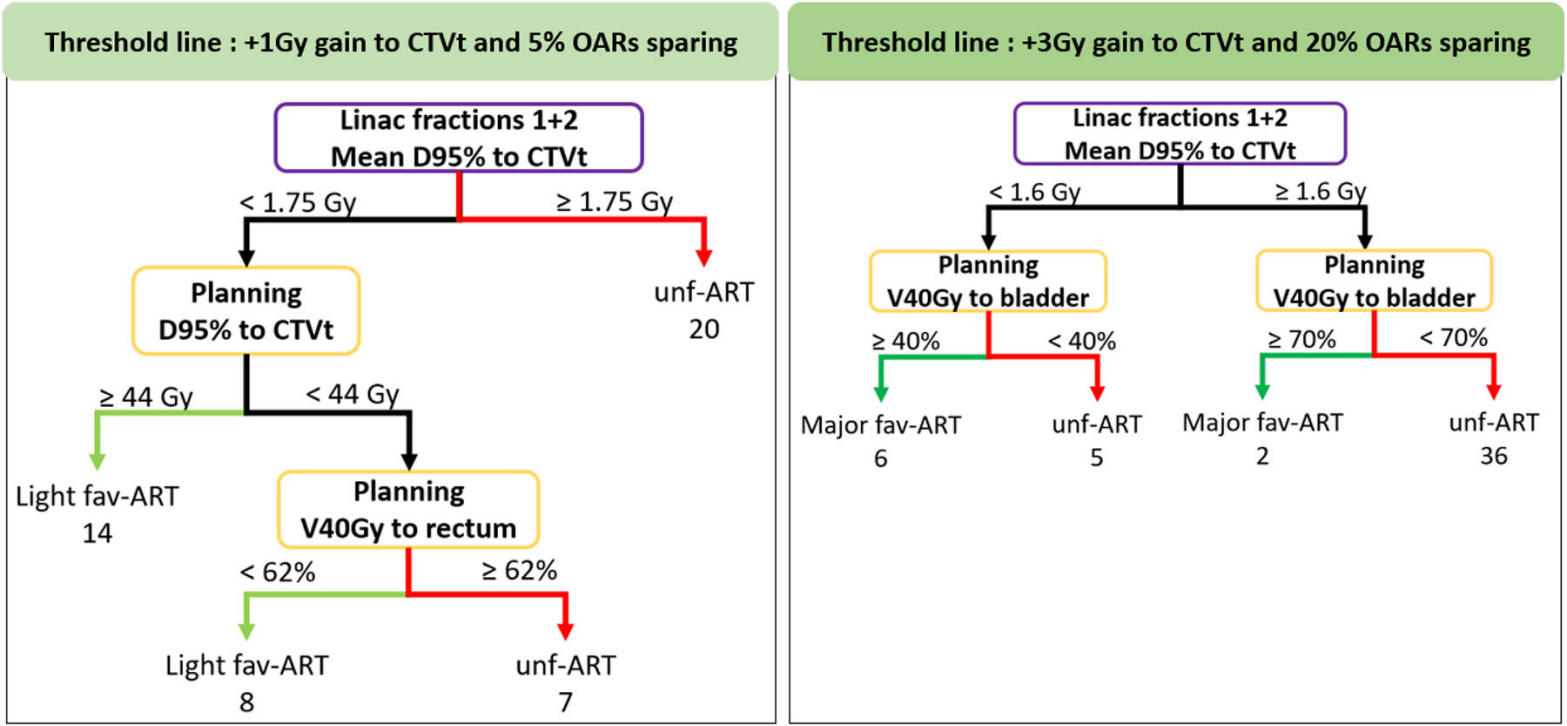
Decision tree using the average dose received to the target (D95%‐CTV) during the first two treatment fractions and from the IB‐CT planning treatment plan. Each branch endpoint shows the predicted strategy (unf‐ART: non‐favorable to ART in red, fav‐ART: favorable to ART in green) with light dosimetric gain (left part) and major dosimetric gain (right part) and the number of individuals assigned to each branch.

Decision trees (Figure 5) were constructed from these variables to predict the membership after two fractions without ART. The first model (unf‐ART and fav‐ART) correctly classified 85.4% of patients (95% confidence interval [0.72,0.94]; *p* < 0.001). It was able to identify most patients needing ART, with sensitivity of 89.5% and specificity of 82.8%. However, 4% of patients requiring ART (fav‐ART) had not been predicted as such, and 10% predicted as fav‐ART with no dosimetric benefit with. The second decision tree, with major dosimetric gains (major fav‐ART), achieved a classification accuracy of 93.8% (confidence interval [0.83,0.99], *p* < 0.05), with a sensitivity of 85.7% and a specificity of 95.1%. This model better captured patients with higher dosimetric gains.

Patients who were misclassified by our predictive models were close to the line separating the two groups and therefore had only a limited dosimetric benefit with fav‐ART.

## 4 DISCUSSION

The goal of this study, in the context of a phase II multicenter clinical trial, was to evaluate an ART workflow for LACC based on a manual selection of the PoD from a library of three different treatment plans based on CTs. This is, to our knowledge, the first multi‐institutional clinical study to combine geometric and dosimetric assessment of PoD‐ART over the entire treatment course. The manual plan selection performed by radiation oncologists was directly compared to an automated coverage‐based strategy, identifying cases where clinical choices aligned with the optimal target coverage, as well as cases where discrepancies occurred, reflecting variability between patients or clinical prioritization of OAR savings. Moreover, we introduced predictive models capable of identifying, early in the treatment, patients most likely to benefit from light or major dosimetric gain from ART. This approach could help to make clinical workflows more efficient and optimize the use of resources by targeting adaptive strategies only to those who would benefit most. In addition to the clinical evaluation of the ART workflow, this study also integrated a deep learning‐based segmentation approach to support large‐scale CBCT analysis.

The nnU‐Net deep‐learning model enabled to automatically generate good or acceptable segmentations for 76.4% of CBCT scans. As far as we know, only four studies have proposed a deep learning‐based segmentation of cervix CBCT, with median reported Dice scores between 0.76 and 0.85.^23^, ^29^, ^30^, ^31^ In our previous work,^23^ we obtained a median Dice score of 0.79 and demonstrated that the segmentation result was able to identify the PoD corresponding to the best coverage for 91.5% of fractions. In the present study, the segmentation evaluation was limited to a visual assessment, since manually delineating the 1217 CBCTs of the present study was not realistic. It resulted in 76.4% of the segmentations being deemed suitable for PoD selection. The subset of poor‐quality segmentations highlighted areas for improvement, challenging cases involving low‐quality images or unusual anatomical variations. These difficulties may also complicate the daily plan selection process for the radiation oncologist. However, our findings indicate that CBCT system variability does not compromise the workflow and study analysis. There was no statistical difference in deep‐learning segmentation accuracy between the Varian and Elekta system.

In this study, an automatic workflow (Cov‐ART) was developed to select the PoD that maximizes the geometrical coverage of the daily CTVt segmented on CBCT images. This method was considered as a theorical reference because its implementation would require automatic segmentation of the daily images followed by calculation of the coverage for each treatment plan. This process is more time consuming which is a crucial point in case of ART. Although it is assumed that optimal coverage would lead to an optimal treatment plan, it does not consider OAR doses, particularly when anatomical displacements reduce the distance between the CTVt and OAR. Nevertheless, this approach demonstrated a concordance of 63.5% with the PoD manually selected by radiation oncologists, with only 3.8% of major discordances. Minor discrepancies can be explained by the high proportion (46.6%) of intermediate bladder plans selected by radiation oncologists. Indeed, in cases of uncertainty, the trial protocol required the radiation oncologist to select the intermediate bladder plan.

In order to limit the impact of nearly identical similar plans, a 5% margin was considered, by including all plans with whose daily CTVt coverage value by the PTV was at least equal to 95% of the coverage value of the PoD from Cov‐ART. Applying a 5% margin, to avoid cases with nearly identical plans, our study found 78.9% concordance, with 19.4% minor discordance and 1.7% major discordance (i.e., 19 cases). Among the 19 major discordances, for 9 cases the CTVt was positioned between multiple plans with no visually superior option, 8 cases involved daily CTVt that were not adequately represented by any of the three treatment plans, for example, CTVt being too anterior or posterior (Figure 2b). Finally, 2 cases involved suboptimal manual choices without justification (Figure 2c). These findings underline the importance of defining an objective and reproductible criterion for treatment plan selection (such as CTVt coverage), especially in cases where inter‐operator variability is significant.

The geometric analysis confirmed that both Manual‐ART and Cov‐ART strategies significantly improved daily CTVt coverage compared to Non‐ART approach, consistent with findings reported in the literature.^10^, ^18^ Cov‐ART strategy slightly outperformed Manual‐ART in terms of median CTVt coverage but also resulted in increased coverage of OARs.

Some ART studies with treatment plan libraries in cervical cancer have been reported in the literature with a limited number of patients, and only three with clinical implementation.^11^, ^32^, ^33^ Compared to plans with margins of 38 mm, ART^10^ significantly spared OARs. For seven adaptive radiotherapy studies for LACC with PoD, dosimetric evaluation was performed with encouraging results both for CTVt and OARs.^11^, ^12^, ^13^, ^15^, ^16^, ^17^, ^18^ Looking at the dose received by 95 or 98% of the CTVt volume, all the studies showed a significant gain in favor of ART (up to +11%^12^). For OARs, only two studies reported a degradation in dose to the bladder: one showed an increase of +2.2 Gy in D50%,^12^ specifically in patients with significant target motion,^15^ while another found no difference in V20Gy for both the bladder and rectum.^33^ Studies with OAR savings^11^, ^13^, ^15^, ^17^, ^18^, ^32^ showed up to 15% to V40Gy for the bladder,^15^ 13% for the rectum and 14 cc for the V30Gy bowel.^32^ Our dosimetric analysis confirmed that PoD‐ART significantly improved D95% of CTVt compared to Non‐ART, increasing the number of fractions compliant with the D95%of CTVt constraint (42.75 Gy), thus ensuring more consistent dose delivery to the CTVt despite anatomical variations. Both Manual‐ART and Cov‐ART improved dose compliance, highlighting the effectiveness of ART in maintaining adequate target coverage despite high inter‐fraction anatomical changes. While the dosimetric results for OARs varied across studies with significant savings^11^, ^12^, ^15^, ^16^, ^17^, ^18^ our findings showed no significant differences in V_40Gy_ to the bladder, peritoneal cavity and rectum between strategies, but Cov‐ART resulted in a slightly higher dose to the rectum compared to Manual‐ART. These findings suggest that a better target coverage can lead to increased OAR doses, highlighting the importance of carefully optimizing safety margins to balance target coverage and OAR sparing. Dosimetric benefits of ART have been demonstrated, its clinical impact remains to be demonstrated and should be explored in future studies with robust quality assurance process. This aligns with studies in favor of ART strategies to optimize dose delivery to the target.

A limitation of this study is the use of direct dose matrix application onto CBCTs without recalculation on synthetic CTs. In the pelvic region, the impact of HU inaccuracies on dose distribution can be considered as limited due to a relative anatomical homogeneity, to limited major weight changes and to the absence of large density gradients (gaz), which allows to assume the invariance of the dose matrix.^25^ Importantly, the same method was applied to all studied approaches (Non‐ART, Manual‐ART and Cov‐ART), limiting the impact of potential bias on the conclusions. Using more advanced techniques (dose warping by deformable image registrations and accumulation) would improve dosimetric accuracy and allow for cumulative dose analysis. Nonetheless, the anatomical deformations observed in our clinical trial were often substantial and irregular for current DIR algorithms to produce reliable and robust results. Future developments in DIR accuracy and image quality may eventually enable such techniques to be applied in pelvic adaptive workflows.

Overall, the dosimetric results reinforce the clinical potential of ART strategies in improving target coverage while maintaining OAR sparing for approximately 45% of patients. Identifying these patients as early as possible in the treatment process would allow for personalized treatment and more efficient allocation of resources. For this purpose, this study introduced a predictive model capable, after 2 days of observation, of identifying patients with dosimetric benefits from Manual‐ART with an accuracy of 85.4%. For the light dosimetric gains, the primary decision node is the delivered dose to the target volume (D95%‐CTV). If the mean D95%‐CTV across the first two fractions exceeds 1.75 Gy (for a 1.8 Gy per fraction prescribed dose), the treatment is considered adequate, and the patient is classified as not requiring ART (unf‐ART). If the D95%‐CTV is below 1.75 Gy, a secondary analysis is performed. When the planned D95%‐CTV is ≥ 44 Gy, the discrepancy between planned and delivered dose suggests target motion during the first fractions, and these patients are classified as fav‐ART. If the planned D95%‐CTV is below 44 Gy, the delivered dose is consistent with the dose planned. These cases likely reflect a coherent treatment delivery relative to the plan, and ART may not be required. To further refine classification, planning the rectal dose is considered. If the V40 Gy to rectum is below 62%, it suggests that the rectum was sufficiently spared in the treatment plans. In such cases, there may be a dosimetric possibility to improve target coverage through ART without exceeding rectal constraints (fav‐ART). Conversely, if V40Gy to rectum is more than 62%, the rectal dose is already near tolerance limits, limiting the potential benefit of ART (unf‐ART). For the major dosimetric gains, patients receiving less than 1.6 Gy on average to the CTVt during the first two fractions were generally considered to require adaptive strategies beyond a simple plan (IB‐CT). Specifically, those classified as ART within this subgroup likely indicate that a single plan was insufficient to cover anatomical variations, emphasizing the need for an expanded plan library with three plans or more, and non‐ART patients need more than three plans or online adaptation. For patients above this dose threshold, bladder sparing on the planning CT was used as a key discriminator: those with good sparing on IB‐CT were classified as non‐ART, while the other patients were considered major fav‐ART. If our model has a 15% misclassification rate, the analysis reveals that these patients are those whose dosimetric benefits were near the decision boundary. For these patients, non‐adaptative treatment remains a clinically acceptable option that does not compromise treatment efficacity. Our model was designed to optimize the use of clinical resources by identifying the patients for whom ART was most beneficial. By focusing on cases with the most significant potential impact, it ensures that resources are directed where they are needed most. These decision trees thus support a dosimetry‐based approach to optimize ART.

This early prediction capability has significant clinical implications, as it could enable personalized treatment and resource optimization. One potential clinical application would be to conduct two series of images under treatment conditions prior to the start of the treatment. This approach would help to determine the optimal strategy between ART and non‐ART for the following treatment course. The choice of reducing the margins of CTV‐PTV can also be discussed depending on the patient, as it can reduce the volume irradiated by an average of 11%.^14^

Our data across the seven centers established that the manual plan selection was relatively effective, typically taking less than 5 min per patient. For easy fractions, this time could be as low as 2–3 min, achieved by using the physician to visually compare the plans library with the daily CBCT. However, when the choice was too difficult, the plan corresponding to an intermediate bladder volume was selected. An optimized and automated workflow incorporating specific metrics (geometrical or dose metrics) would be: perform the deep learning segmentation on daily CBCT (estimated time 2 min), calculate the coverage of target and OARs (estimated time 2 min) and automatically identify the most suitable plan (estimated time 15 s). This process would require only a quick verification, ensuring low time‐per‐fraction.

Both our geometric and dosimetric analyses were strongly based on the results of segmentation. As a result, 24% of the data had to be excluded from the analysis. To address these challenges, recent advancements in deep learning have focused on enhancing CBCT image quality. One promising approach is synthetic CT, which has shown potential to produce high‐quality, artefact‐corrected CBCT images, and improving the robustness of segmentation models in ART workflows.^34^, ^35^ Moreover, the manual PoD selection process, while effective in many cases, was subject to inter‐observer variability^13^ especially in cases of complex deformations or poor image quality. While geometric coverage was the important criterion for geometric strategies comparison as recommended in the literature^11^, ^30^ dosimetric analyses are influenced by the methodology employed for dose calculation. This study applied dose matrix directly to the CBCT for each fraction. However, dosimetric evaluations could be achieved using pseudo‐CT dose recalculation,^12^ density assignment^18^, ^36^ or deformable registration for dose accumulation.^13^

Lastly, although the PoD‐ART approach utilizing multiple planning CTs has been implemented, it has demonstrated certain limitations in particular with tumor shrinkage and large CTVt motion. To address these limitations, more advanced strategies have been suggested to improve the libraries. For instance, an “evolutive library” approach has been introduced, which incorporates CBCT anatomies into the library when the CTVt shape deviates from those in the library^18^ avoiding rescheduling to optimize new treatment plan libraries. Another proposed method could be to include modeled anatomies obtained from a population analysis.^37^ Additionally, these strategies to provide meaningful clinical benefits, it should always be implemented based on quantifiable and objective selection criteria, rather than subjective ones, similar to the principles guiding online adaptive radiotherapy.

Recent advances in radiotherapy, with online radiation therapy (oART) using CBCT or MR images enable dose reduction to OARs, while respecting the target prescription dose by a daily treatment plan optimization, and may decrease toxicities.^38^, ^39^, ^40^, ^41^, ^42^, ^43^, ^44^, ^45^ Reduced margins are now primarily used to account for intra‐fraction motion, as inter‐fraction variations are managed through daily planning based on daily image. This remains crucial, especially with oART, which requires longer treatment sessions (contouring of organs and optimization of plan) that may increase the risk of organ motion during irradiation. Moreover, daily contours are often generated through contour propagation, with 10%–20% requiring manual adjustments,^39^, ^41^, ^42^, ^46^ which necessitates significant resources. Thus, future work has to consider comparison between PoD‐ART and oART approaches, as both rely on similar methodological considerations. Personalizing treatment based on organ motion remains a key challenge, highlighting the importance of patient selection criteria and the adaptation of treatment margins to account for observed motion patterns.

## 5 CONCLUSION

PoD manually selected by physicians achieved 63.5% agreement with optimal plans based on geometric target coverage. Geometric and dosimetric analyses showed an overall dosimetric benefit to the target with the manual PoD strategy compared to Non‐ART, but heterogeneous results between patients. As a result, a sub‐population with benefit from manual‐ART was identified with an accuracy of 85.4% for light dosimetric gain and 93.8% for major dosimetric gain, using decision tree with the CTVt coverage from the first two treatment fractions and planning dosimetric indices, enabling the selection of the most suitable treatment strategy between Non‐ART and Manual‐ART for each patient.

## AUTHOR CONTRIBUTIONS

Caroline Lafond, Anaïs Barateau, Renaud de Crevoisier and Antoine Simon contributed to the conception and design of the study. Delphine Lebret, Julie Leseur, Diane Chan Sock Line performed experiments, evaluation, and analysis. Julie Leseur, Karine Peignaux, Nathalie Mesgouez‐Nebout, Magali Le Blanc‐Onfroy, Chantal Hanzen, Nedjla Allouache, Sophie Renard‐Oldrini, Florence Le Tinier provided clinical imaging data. Delphine Lebret wrote the first draft of the manuscript. All authors contributed to the manuscript revision, read and approved the submitted version.

## CONFLICT OF INTEREST STATEMENT

The authors declare no conflicts of interest.

## ETHICS STATEMENT

This study was carried out in compliance with the principles of the Declaration of Helsinki and applicable local regulations. It received approval from the regional research ethics committee (Comité de Protection des Personnes, Ouest V, Rennes, ARCOL 2015‐02‐45‐01). Written informed consent was obtained from all participants prior to their inclusion in the study.
